# Nasal intermittent positive pressure ventilation in neonates with grade 3 bronchopulmonary dysplasia

**DOI:** 10.1038/s41372-025-02472-1

**Published:** 2025-11-17

**Authors:** Mark F. Weems, Vineet Lamba, Sandeep Chilakala, L. Brooke Murdock, Divya Rana, Rishika Sakaria, Parul Zaveri, Rangasamy Ramanathan

**Affiliations:** 1 Department of Pediatrics, Division of Neonatology, University of Tennessee Health Science Center, Memphis, TN USA; 2 Le Bonheur Children’s Hospital, Memphis, TN USA; 3 Sutter Medical Center, Sacramento, CA USA; 4 Lakeland Regional Medical Center, Lakeland, FL USA; 5 Department of Pediatrics, Division of Neonatology, Cedars Sinai Guerin Children’s, Cedars Sinai Medical Center, Los Angeles, CA USA; 6 Department of Pediatrics, Division of Palliative Care, University of Tennessee Health Science Center, Memphis, TN USA; 7 Department of Pediatrics, Division of Cardiology, University of Tennessee Health Science Center, Memphis, TN USA

**Keywords:** Respiratory tract diseases, Paediatrics

## Abstract

**Objective:**

We describe a novel strategy of nasal intermittent positive pressure ventilation (NIPPV) to support patients with Grade 3 bronchopulmonary dysplasia (BPD).

**Study design:**

This is a retrospective study of Grade 3 BPD patients treated with NIPPV and discharged from a single center from January 2020 to May 2024. Patients were grouped into discharged without tracheostomy vs with tracheostomy. Groups were assessed for clinical differences, and the NIPPV strategy is described.

**Results:**

There were 28 non-tracheostomy and 17 tracheostomy patients. There were no differences in gestational age, birthweight, or respiratory severity score at key dates. Tracheostomy patients were more likely to have subglottic stenosis (53% vs 3.6%, *p* = 0.0001) and were older at discharge home [median 447 (411–479) vs 252 (184–309) days, *p* < 0.0001].

**Conclusion:**

A subset of Grade 3 BPD patients can be supported with NIPPV. The non-tracheostomy group had decreased length of stay compared to the tracheostomy group.

## Introduction

Recent advancements in neonatology have led to improved survival among extremely preterm infants. Improved survival, however, is associated with increased morbidity, and there has been an increase in patients with bronchopulmonary dysplasia (BPD) [1]. These patients often have a prolonged hospital course, and those with Grade 3 BPD who remain intubated at 36–44 weeks postmenstrual age (PMA) are frequently treated with tracheostomy to support long-term mechanical ventilation (LTMV) [2, 3]. While a tracheostomy in patients with BPD is generally temporary, discharge to home with a tracheostomy and ventilator increases complexity and requires significant resources [4]. In our community, the lack of a long-term care facility and a persistent shortage of specialized pediatric home nursing has contributed to an increase in hospital length of stay for LTMV patients.

The use of nasal intermittent positive pressure ventilation (NIPPV) has increased as a primary non-invasive mode or following a period of invasive mechanical ventilation in preterm infants with respiratory distress syndrome and has been shown to increase the rate of successful extubation [5, 6]. However, if extubation is not possible before 36 weeks PMA, there is growing clinical consensus that shifting to a chronic-phase ventilation strategy helps to optimize late outcomes [7]. The use of NIPPV during the chronic phase of established BPD beyond 36 weeks PMA has not been well described, and two recent observational studies of severe BPD patients showed that NIPPV is the least commonly used mode of noninvasive support [8, 9].

To decrease tracheostomy use and reduce length of stay, we started using NIPPV to facilitate extubation in patients with Grade 3 BPD who are not yet able to tolerate nasal continuous positive airway pressure (CPAP) or nasal cannula. In this study, we aim to describe a novel strategy of NIPPV used as a bridge between chronic-phase ventilation and CPAP in patients with Grade 3 BPD and to explore differences between those who were discharged without tracheostomy and those who were discharged after tracheostomy in a single Level IV NICU.

## Materials/subjects and methods

This is a retrospective observational study at a single all-referral Level IV NICU. Patients were included if they were born prior to 32 weeks’ gestation, had a diagnosis of Grade 3 BPD [ie, they remained intubated at 36 weeks postmenstrual age (PMA)] [3], and were discharged from January 2020 to May 2024. Patients were excluded if there was no extubation attempt after 36 weeks PMA. All patients were outborn and transferred to the Level IV NICU with variable severity of BPD. Patients were identified prospectively within an ongoing BPD quality improvement (QI) project focused on standardized guidelines for invasive mechanical ventilation support and extubation criteria for patients with established BPD. Patients were divided into two groups by the primary outcome of discharge with or without tracheostomy.

NIPPV was delivered via an appropriately sized RAM Cannula® (NEOTech, Chatsworth, CA) connected to a ventilator. RAM Cannula® size was selected so that the cannula fills approximately 80% of the nares, generally with the orange or yellow marked cannula. Management decisions regarding ventilator model and settings were left to the discretion of the treating physician. During the study period, two ventilators were used for NIPPV. The Dräger V-500 (Drägerwerk AG & Co. KGaA, Lübeck, Germany) delivered NIPPV in the PC-CMV mode, and the Servo-I (Getinge, Solna, Sweden) delivered NIPPV in the NIV-PC mode. Local NIPPV guidelines (supplement) recommended post-extubation peak inspiratory pressure (PIP) in the range of 30–35 cmH_2_O with a maximum recommended pressure of 45 cmH_2_O. The recommendation for positive end expiratory pressure (PEEP) was 2–3 cmH_2_O above the previous invasive PEEP with a maximum of 12 to 14 cmH_2_O. Both PIP and PEEP were set higher than the pre-extubation pressures to compensate for leak. Our guidelines recommended NIPPV rate of 40 (range 30–50) with an inspiratory time of 0.5–0.6 s (maximum of 1 s). Generally, PIP was weaned to approximately 20 cmH_2_O before decreasing rate or PEEP. When minimal PIP was reached, the guidelines suggested weaning rate by 10 every 6 h until the patient was on CPAP.

Within the BPD QI project, clinical data were collected daily from January 2020 to August 2021 and weekly from August 2021 through May 2024. Data were collected and managed using REDCap electronic data capture tools hosted at the University of Tennessee Health Science Center [10, 11]. Additional data were extracted from the electronic medical records. Data were imported into Stata 18 (StataCorp LLC, College Station, TX) for analysis. Wilcoxon rank sum test and test of proportions were used to compare groups. A *p* value of <0.05 was considered statistically significant.

Successful extubation was defined as each patient’s final extubation prior to discharge excluding intubations specifically for procedures under sedation. A failed extubation was defined as an extubation in a patient older than 36 weeks PMA who was supported with NIPPV and later required intubation due to respiratory failure at some point during the initial hospitalization. Respiratory severity score (RSS) for intubated patients was calculated by the product of fraction of inspired oxygen (FiO_2_) and the mean airway pressure (MAP). For patients on NIPPV, the RSS was factored at 75% to adjust for leak [12]. Airway anomaly findings were based on visualization by the otorhinolaryngologist or at least 50% collapse of airways visualized on dynamic computed tomography of the chest [13–15].

## Results

Fifty-seven patients met inclusion criteria. Twelve patients were excluded because they were never extubated after 36 weeks PMA: one had a known diagnosis of subglottic stenosis and eleven never met extubation criteria, among whom seven died prior to tracheostomy, two died after tracheostomy, and two survived with tracheostomy. Forty-five patients were available for analysis. The median gestational age was 25 weeks, and median birth weight was 680 g. Patients were admitted to our referral center at a median PMA of 34 weeks or 60 days of age. Detailed patient characteristics are presented in Table 1. When comparing markers of severity of illness, there were no significant differences in RSS at 36 weeks PMA, necrotizing enterocolitis (NEC) prior to 36 weeks PMA, patent ductus arteriosus (PDA) device closure, or severe retinopathy of prematurity (ROP) needing treatment.

**Table 1:** Patient characteristics.

	All patients	No Tracheostomy	Tracheostomy	*p* value
Patients, n	45	28	17	
Gestational age, weeks, median (IQR)	25 (24–26)	25 (24–27)	24 (24–26)	0.11
Birthweight, g, median (IQR)	680 (550–810)	708 (590–825)	590 (530–750)	0.32
Sex				
Male, n (%)	23 (51)	16 (57)	7 (41)	0.3
Female, n (%)	22 (49)	12 (43)	10 (59)	
Age at admission, days, median (IQR)	60 (21–120)	40 (15–111)	83 (41–120)	0.42
PMA at admission, weeks, median (IQR)	34 (29–41)	31 (29–41)	38 (31–41)	0.56
First RSS at 36 weeks PMA or admission, median (IQR)*	5.43 (3.79–7.67)	5.42 (3.78–7.53)	5.43 (4.09–8.76)	0.61
Surgical NEC prior to 36 weeks PMA, n (%)	7 (16)	5 (18)	2 (12)	0.58
PDA Device closure prior to 36 weeks PMA, n (%)	13 (29)	8 (29)	5 (29)	0.95
ROP requiring bevacizumab prior to 36 weeks PMA, n (%)	1 (2.2)	1 (3.6)	0	0.33

^*^4 patients with first RSS recorded ≥38 weeks PMA.

*PMA* Postmenstrual age, *NEC* Necrotizing enterocolitis, *ROP* Retinopathy of prematurity, *PDA* Patent ductus arteriosus

Patient outcomes are presented in Table 2. The RSS at 44 weeks PMA and prior to event (i.e. extubation or tracheostomy placement) was comparable between both groups. There were no patient deaths in either group. The tracheostomy group was significantly more likely to have airway anomalies, mostly subglottic stenosis. Sixteen patients were diagnosed with an airway anomaly. Three patients had airway malacia diagnosed with computed tomography. The remaining patients had airway anomalies diagnosed by rigid bronchoscopy, including one case of vocal cord paralysis (tracheostomy group) and one case of supraglottic granuloma (no tracheostomy). Three patients in the tracheostomy group had clinical suspicion of airway anomaly but had a normal airway evaluation by bronchoscopy. In the no tracheostomy group, 21 (75%) were discharged on supplemental oxygen and seven (25%) were discharged in room air. Whereas in the tracheostomy group, 12 (71%) patients were on mechanical ventilation, 2 (12%) were on continuous positive airway pressure (CPAP), and three (18%) were on humidified oxygen without positive pressure. The no tracheostomy group had a significantly shorter length of hospital stay.

**Table 2:** Patient outcomes.

	All patients (*n* = 45)	No tracheostomy (*n* = 28)	Tracheostomy (*n* = 17)	*p* value
Patients with at ≥1 failed extubation after 36 weeks PMA, n (%)	28 (62)	11 (39)	17 (100)	<0.001
Age at successful extubation or trach, days, median (IQR)	146 (110–183)	122 (91–159)	183 (168–212)	<0.001
PMA at successful extubation or trach, weeks, median (IQR)	46 (41–51)	43 (39–47)	51 (50–53)	<0.001
RSS prior to event, median (IQR), *n* = 25	3.5 (2.74–4.24)	3.5 (2.8–4.2)	3.86 (2.29–4.29)	0.99
RSS at 44 weeks PMA, median (IQR)	3.64 (1.8–5.13)	3.74 (1.54–5.16)	3.02 (2.49–4.82)	0.73
Pulmonary hypertension treated during hospitalization, n (%)	14 (31)	6 (21)	8 (47)	0.074
Airway anomaly, n (%)	16 (36)	4 (14)	12 (71)	0.0001
Airway Malacia, n (%)	4 (8.9)	2 (7.1)	2 (12)	0.6
Subglottic stenosis, n (%)	10 (22)	1 (3.6)	9 (53)	<0.001
Age at hospital discharge, days, median (IQR)	301 (214–429)	252 (184–309)	447 (411–479)	<0.001
PMA at hospital discharge, weeks, median (IQR)	70 (57–86)	61 (51-71)	88 (84–93)	<0.001

*PMA* Postmenstrual age, *RSS* Respiratory severity score.

**Fig. 1:**
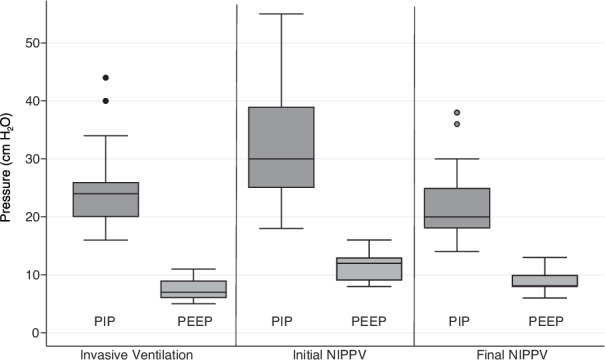
Pre-extubation PIP [24 (20–26) cmH_2_O], pre-extubation PEEP [7 (6–9) cmH_2_O], initial NIPPV PIP [30 (25–39) cmH_2_O] after extubation, initial NIPPV PEEP [12 (9–13) cmH_2_O] after extubation, and final PIP [20 (18–25) cmH_2_O] before transition to CPAP, and final PEEP [8 (8–10) cmH_2_O] before transition to CPAP. PIP positive inspiratory pressure, PEEP positive end expiratory pressure, CPAP continuous positive airway pressure, NIPPV nasal intermittent positive pressure ventlation.

**Fig. 2:**
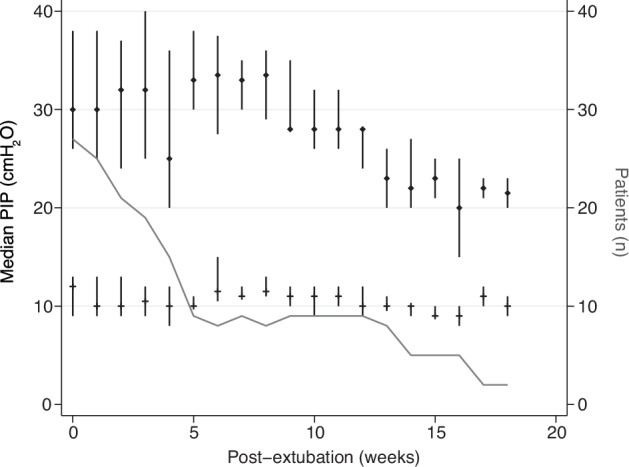
Post-extubation median (IQR) PIP and PEEP (cmH_2_O) over time after extubation (weeks). Solid line represents the number of patients who remain on NIPPV each week. One patient excluded because he remained on PIP approximately 45 and PEEP approximately 14 for >20 weeks at which point he was changed to high-flow nasal cannula 10 lpm. PIP peak inspiratory pressure, PEEP positive end expiratory pressure, NIPPV nasal intermittent positive pressure ventilation.

**Fig. 3:**
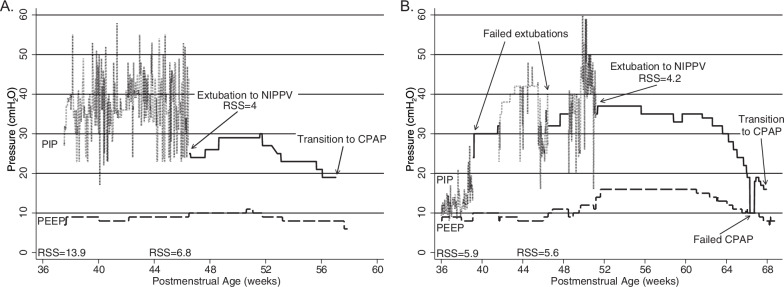
PIP and PEEP for two example patients extubated from volume-targeted ventilation with variable PIP to NIPPV. **A** Extubation to NIPPV at 46 weeks PMA. Initial NIPPV PIP was set too low and increased over 3 weeks before the infant was stable and weaning was possible. **B** Successful extubation to NIPPV at 51 weeks PMA after failed extubations at 39 and 46 weeks PMA. PIP peak inspiratory pressure, PEEP positive end expiratory pressure, NIPPV nasal intermittent positive pressure ventilation, PMA postmenstrual age, RSS respiratory severity score, CPAP continuous positive airway pressure.

Figure 1 illustrates median PIP and PEEP at three phases of NIPPV weaning. Pre-extubation PIP was a median 24 cmH_2_O (IQR 20–26), and pre-extubation PEEP was a median 7 cmH_2_O (IQR 6–9). After extubation and stabilization on NIPPV, the initial PIP was a median 30 cmH_2_O (IQR 25-39), and the initial PEEP was a median 12 cmH_2_O (IQR 9-13). Before the transition from NIPPV to CPAP, the final PIP was a median 20 cmH_2_O (IQR 18–25), and the final PEEP was a median 8 cmH_2_O (IQR 8–10). Median duration of NIPPV was 35 days (IQR 14–53) before transition to CPAP. Figure 2 illustrates post-extubation PIP and PEEP over time after extubation. Median NIPPV rate was 35 (30-40) breaths per minute and inspiratory time was 0.6 (0.5–0.6) seconds; these variables did not change significantly over the weaning period. Figure 3 highlights two case examples demonstrating changes in PIP and PEEP over time during NIPPV mode.

## Discussion

In this observational study, we report on a cohort of infants with Grade 3 BPD who were extubated to NIPPV after 36 weeks PMA. Sixty-two percent of patients were discharged home without tracheostomy, and 38% had tracheostomy. While NIPPV is now widely used by neonatologists, NIPPV for patients with Grade 3 BPD as reported in this study has not previously been described for use in a population whose lung disease is “sufficiently abnormal that attempts at extubation to nasal CPAP are neither feasible nor desirable.”[16]. We showed that there is a subset of Grade 3 BPD patients who can be successfully supported with NIPPV and weaned to minimal support for discharge rather than proceeding with tracheostomy [3, 17].

Failed extubation attempts beyond 36 weeks PMA were common in both groups, indicating a significant level of lung disease was present among these infants. In the no tracheostomy group, 39% of infants had a failed extubation after 36 weeks; all patients in the tracheostomy group also failed at least one extubation after 36 weeks. These extubations are in addition to any that may have been attempted prior to 36 weeks PMA. This illustrates that all infants in this study met extubation criteria at some point after 36 weeks PMA. While tracheostomy may sometimes be a marker of disease severity, it is also a therapeutic choice dependent on the biases of the clinicians and parents. Our finding that respiratory severity score at 36 weeks or 44 weeks PMA was not associated with tracheostomy indicates there are unmeasured factors beyond lung disease that are associated with tracheostomy use. We found airway issues were more common in the tracheostomy group; specifically, more patients in this group were found to have subglottic stenosis. However, unless there is a specific airway evaluation, subglottic stenosis may not be diagnosed until the time of tracheostomy or later. These findings suggest that earlier efforts to diagnose subglottic stenosis may better identify patients who are at higher risk for tracheostomy than relying only on the severity of respiratory disease or a history of failed extubations. Both chronological age and PMA were older at the time of tracheostomy compared to the time of successful extubation in the no tracheostomy group; this finding is expected in a clinical practice that moves to tracheostomy after multiple failed extubation attempts and tries to avoid tracheostomy whenever possible.

We also found a significantly longer length of stay in the tracheostomy group. There are several known and suspected factors that may explain the length of hospital stay differences between the groups. While this may reflect that patients who are able to be managed with NIPPV may be generally less sick than those who ultimately received a tracheostomy, we show that markers of lung disease severity at key time points are similar between the groups suggesting there may be clinical differences that occur after the patient is successfully extubated or receives a tracheostomy. In our center, management of the patients prior to extubation or tracheostomy is standardized, but there is divergence of practice after extubation or tracheostomy. The no tracheostomy group continues to be managed by the neonatologists until discharge while the tracheostomy group is comanaged by the pulmonologists prior to discharge. Differences of practice between these specialists along with differences in discharge criteria may affect the frequency of ventilator weaning and as well as clinical goals for each patient. Furthermore, we also hypothesis that adequate NIPPV support may have a positive effect on lung development relative to continued mechanical ventilation and allow the NIPPV patient to progress through the phases of BPD at a faster pace, allowing improved developmental care. Irrespective of practice differences and the potential effect of NIPPV on lung development, length of stay in our community is influenced by significant barriers to support patients with tracheostomies in the home setting. The identification of stable care providers, home training, addressing home safety issues, and securing home nursing each adds additional delays to discharge in patients with a tracheostomy.

There are little data to guide respiratory support strategies in patients with Grade 3 BPD, but a best-practice guideline was published by the BPD Collaborative in 2017 suggesting a strategy for invasive ventilator management that differs from traditional strategies used in neonatology [16]. While many centers have adopted the described low-rate, high-tidal volume strategy for chronic-phase invasive mechanical ventilation, there is no consensus for tracheostomy placement or post-extubation support [8, 18]. It has been suggested that extubation to nasal CPAP is preferred, but NIPPV has consistently been shown to be associated with higher rates of extubation success compared to nasal CPAP [5–7]. Although NIPPV may better support extubation success, NIPPV has not been well studied in the severe BPD population, and it was rarely used in a study of respiratory support modes across the BPD Collaborative, with nasal cannula or nasal CPAP being much more common [8]. In a more recent study with data from the Children’s Hospitals Neonatal Database, NIPPV was used more frequently to support patients with severe BPD at 36 weeks PMA but still less than half as often as CPAP [9].

Nasal CPAP supports the infant after extubation by reducing airway resistance and preventing volume loss and atelectasis [19]. In the case of Grade 3 BPD, however, the infant may be extubated from relatively high tidal volume support which cannot be replicated with CPAP [16]. Extubating to NIPPV may overcome this shortfall by increasing tidal volume for supported breaths and thereby improving minute ventilation [19]. However, there are significant differences in NIPPV strategies, pressure-delivery systems, and nasal interfaces which make it difficult to interpret results from published studies [5].

We utilized the RAM Cannula for its ease of use and decreased rates of nasal injury compared to other devices [20]. Although the RAM Cannula is approved as an oxygen delivery device, clinicians have adapted its use for NIPPV, typically for treatment of respiratory distress syndrome (RDS), and there are no published recommendations for the use of NIPPV or RAM Cannula for patients with Grade 3 BPD [20, 21]. Our NIPPV strategy was developed by adapting what has been described for preterm infants during the acute phase of respiratory disease. Typically, NIPPV strategies during this phase recommend PIP 20–30 cmH_2_O and PEEP 5–8 cmH_2_O [19]. The ongoing DIVA Trial (NCT05446272), for example, recommends PIP only 10 cmH_2_O above a PEEP in the range of 5–10 cmH_2_O, recommended rate is 30 breaths per minute, and inspiratory time is 0.3 to 0.5 s (D. Matlock, MD, University of Arkansas for Medical Sciences, email correspondence, September 24, 2025) [22]. Our strategy for NIPPV using a median starting PIP of 30 cmH_2_O and PEEP of 12 cmH_2_O recognizes that patients with established BPD often have lung disease complicated by airway obstruction, high airway resistance, and regional heterogeneity and benefit from larger tidal volumes, increased PEEP, and longer inspiratory times compared to typical respiratory support during the first weeks of respiratory disease [16].

We chose relatively high PIP and PEEP values to address the variable leak associated with RAM Cannula. Under optimal conditions, pressure delivered through an appropriately sized RAM cannula is approximately 75% of the set pressure [12, 23]. Therefore, it stands to reason that NIPPV PIP and PEEP can safely be set at 133% of the invasive settings without fear of worsening barotrauma, and pressure delivery is likely significantly lower in most real-life circumstances. We typically set PIP 5–6 cmH_2_O above the previous invasive PIP and PEEP 2–3 cmH_2_O above the previous invasive PEEP.

**Fig. 4:**
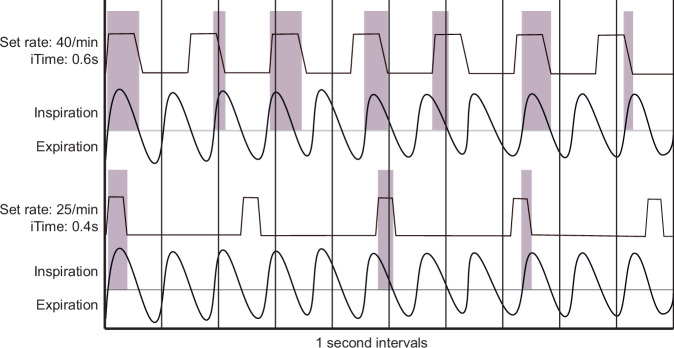
Illustration of non-synchronized overlap with a hypothetical spontaneous respiratory rate of 65 bpm over 10 s. Ventilator breaths are effective only when they overlap with spontaneous inspiration as marked in the shaded area. With a ventilator set rate of 40 bpm and inspiratory time of 0.6 s, 7 of 11 breaths are at least partially supported. This reaches the >60% threshold for effective NIPPV [27]. In this model, 2.6 of the 10 s are spent in ventilator-supported inspiration (inspiration-to-expiration ratio 1:3). With a set rate of 25 bpm and inspiratory time of 0.4 s, only 3 of 11 breaths are partially supported. This is ineffective NIPPV because supported breaths are <60% of spontaneous breaths. Here, only 0.75 s is spent in ventilator-supported inspiration. bpm breaths per minute, NIPPV nasal intermittent positive pressure ventilation.

Non-synchronized NIPPV delivers time-cycled, pressure-controlled breaths. Ventilator breaths delivered during patient exhalation or during a period of apnea are not transmitted to the alveoli [24–26]. Adopting a low-rate NIPPV strategy, similar to what is used in chronic-phase invasive ventilation, has been shown to be no different than CPAP. This is likely because too few patient breaths coincide with ventilator breaths as the benefits of NIPPV on respiratory effort occur when at least 60% of infant breaths are supported ([27, 28]). Therefore, we used the inspiratory time and rate to allow for at least 60% overlap between ventilator breaths and infant inspiration (Fig. 4). To accomplish this goal, our practice is to start NIPPV with a rate of 40 (range 30–50) and inspiratory time of 0.5–0.6 s, and the rate is only decreased to less than 30 at the time of transition to nasal CPAP. Although this strategy may seem to conflict with the prolonged exhalation time commonly applied to chronic-phase invasive mechanical ventilation strategies in patients with Grade 3 BPD patients, the lack of pressure transmission to the alveoli during patient exhalation limits the effective inspiratory time and allows for adequate exhalation even in the presence of a high ventilator set rate.

After extubation to NIPPV, settings may be adjusted as needed to optimize patient comfort and support oxygenation. There are then several weeks of stability during which the clinical team assesses oxygen needs and changes in growth velocity. When markers of oxygenation, ventilation, and growth indicate weaning is possible, we recommend weaning PIP one to two times weekly for a total of approximately 3 cmH_2_O each week until the patient is ready for transition to CPAP. In cases of clinical deterioration after extubation, PIP, PEEP, or inspiratory time is increased as needed, and intubation is reserved for patients who do not improve on higher settings.

This study is limited by its observational design and single-center experience. Although most demographic variables and RSS were similar between the groups, there was no true control group. The increased rate of subglottic stenosis in the tracheostomy group suggests there is heterogeneity and phenotype differences among patients with Grade 3 BPD that may not be recognized until late in the patient’s course. Airway anomalies, for example, may not be diagnosed without a specific airway evaluation or intervention. However, airway evaluations were not performed in all patients, and some patients with airway anomalies may have been missed if there was no clinical suspicion. The high rate of positive pressure support at discharge in the tracheostomy group suggests there may have been an unrecognized severity of lung disease that differed from the no tracheostomy group. However, this may also reflect different clinical strategies between the neonatologists who continue to manage the NIPPV patients until discharge and the pulmonologists who begin to manage the ventilator when the tracheostomy patient has reached post-operative stability. It also reflects the fact that we have a mechanism to discharge patients from this NICU on positive pressure support after tracheostomy while NIPPV patients must be weaned off positive pressure prior to discharge. There also may be other clinical differences that will become relevant with a larger study population. The described NIPPV strategy was selected based on observation of clinical practice. Individual patient care was determined by the clinical team, and there may be alternate strategies that are more effective. Although our practice evolved to use NIPPV to support extubation in patients who were not adequately supported with nasal CPAP, we do not have any comparison groups to test the effect of different non-invasive support modes. Due to data collection limitations, there may have been ventilator changes that occurred between data collection times and were not captured. Because our center is an all-referral NICU, pre-admission data that may impact respiratory outcomes such as duration of intubation and steroid exposure is not consistently available, and pre-admission management likely has a significant impact on outcomes. It will also be important to understand long-term respiratory and developmental outcomes associated with NIPPV, but we currently have no data beyond hospital discharge.

This study shows that a subset of Grade 3 BPD patients can successfully be extubated from invasive mechanical ventilation using non-synchronized NIPPV mode delivered via the RAM Cannula with a strategy utilizing PIP and PEEP higher than previously described and a rate 30–50. In this study, those who were successfully extubated to NIPPV and discharged without tracheostomy had decreased length of hospital stay compared to those discharged with tracheostomy and LTMV. We also identify that subglottic stenosis is a significant comorbidity among those infants who receive a tracheostomy. Additional research is needed to determine optimal NIPPV support strategies and patient selection.

## Supplementary information

Center for Lung Development Clinical Practice Guidelines

## Data Availability

De-identified data is available from the corresponding author upon request.

## References

[1] Lui K, Lee SK, Kusuda S, Adams M, Vento M, Reichman B, et al. Trends in outcomes for neonates born very preterm and very low birth weight in 11 high-income countries. J Pediatr. 2019;215:32–40.e14. 31587861 10.1016/j.jpeds.2019.08.020

[2] Han SM, Watters KF, Hong CR, Edwards EM, Knell J, Morrow KA, et al. Tracheostomy in very low birth weight infants: a prospective multicenter study. Pediatrics. 2020;145:e20192371. 10.1542/peds.2019-2371 32098788

[3] Jensen EA, Dysart K, Gantz MG, McDonald S, Bamat NA, Keszler M, et al. The diagnosis of bronchopulmonary dysplasia in very preterm infants. an evidence-based approach. Am J Respir Crit Care Med. 2019;200:751–9. 30995069 10.1164/rccm.201812-2348OC PMC6775872

[4] Upadhyay K, Vallarino DA, Talati AJ. Outcomes of neonates with tracheostomy secondary to bronchopulmonary dysplasia. BMC Pediatr. 2020;20:414. 32873254 10.1186/s12887-020-02324-1 PMC7459155

[5] Lemyre B, Deguise MO, Benson P, Kirpalani H, De Paoli AG, Davis PG. Nasal intermittent positive pressure ventilation (NIPPV) versus nasal continuous positive airway pressure (NCPAP) for preterm neonates after extubation. Cochrane Database Syst Rev. 2023;7:CD003212. 37497794 10.1002/14651858.CD003212.pub4 PMC10374244

[6] Lemyre B, Deguise MO, Benson P, Kirpalani H, Ekhaguere OA, Davis PG. Early nasal intermittent positive pressure ventilation (NIPPV) versus early nasal continuous positive airway pressure (NCPAP) for preterm infants. Cochrane Database Syst Rev. 2023;7:CD005384. 37466143 10.1002/14651858.CD005384.pub3 PMC10355255

[7] Sindelar R, Shepherd EG, Agren J, Panitch HB, Abman SH, Nelin LD, et al. Established severe BPD: is there a way out? Change of ventilatory paradigms. Pediatr Res. 2021;90:1139–46. 34012026 10.1038/s41390-021-01558-8

[8] McKinney RL, Napolitano N, Levin JJ, Kielt MJ, Abman SH, Guaman MC, et al. Ventilatory Strategies in Infants with Established Severe Bronchopulmonary Dysplasia: A Multicenter Point Prevalence Study. J Pediatr. 2022;242:248–52.e241. 34710394 10.1016/j.jpeds.2021.10.036 PMC10478127

[9] Kielt MJ, Zaniletti I, Lagatta JM, Padula MA, Grover TR, Porta NFM, et al. Liberation from Respiratory Support in Bronchopulmonary Dysplasia. J Pediatr. 2025;282:114390. 10.1016/j.jpeds.2024.114390 39521174

[10] Harris PA, Taylor R, Minor BL, Elliott V, Fernandez M, O’Neal L, et al. The REDCap consortium: Building an international community of software platform partners. J Biomed Inf. 2019;95:103208. 10.1016/j.jbi.2019.103208 PMC7254481 31078660

[11] Harris PA, Taylor R, Thielke R, Payne J, Gonzalez N, Conde JG. Research electronic data capture (REDCap)-a metadata-driven methodology and workflow process for providing translational research informatics support. J Biomed Inf. 2009;42:377–81. 10.1016/j.jbi.2008.08.010 PMC2700030 18929686

[12] Iyer NP, Chatburn R. Evaluation of a nasal cannula in noninvasive ventilation using a lung simulator. Respir Care. 2015;60:508–12. 25492958 10.4187/respcare.03560

[13] Wallis C, Alexopoulou E, Anton-Pacheco JL, Bhatt JM, Bush A, Chang AB, et al. ERS statement on tracheomalacia and bronchomalacia in children. Eur Respir J. 2019;54:1900382. 10.1183/13993003.00382-2019 31320455

[14] Lee KS, Sun MRM, Ernst A, Feller-Kopman D, Majid A, Boiselle PM. Comparison of dynamic expiratory CT with bronchoscopy for diagnosing airway malacia: a pilot evaluation. Chest. 2007;131:758–64. 17356090 10.1378/chest.06-2164

[15] Tan JZ, Crossett M, Ditchfield M. Dynamic volumetric computed tomographic assessment of the young paediatric airway: Initial experience of rapid, non-invasive, four-dimensional technique. J Med Imaging Radiat Oncol. 2013;57:141–8. 23551770 10.1111/1754-9485.12009

[16] Abman SH, Collaco JM, Shepherd EG, Keszler M, Cuevas-Guaman M, Welty SE, et al. Interdisciplinary care of children with severe bronchopulmonary dysplasia. J Pediatr. 2017;181:12–28.e11. 27908648 10.1016/j.jpeds.2016.10.082 PMC5562402

[17] Gibbs K, Jensen EA, Alexiou S, Munson D, Zhang H. Ventilation strategies in severe bronchopulmonary dysplasia. Neoreviews. 2020;21:e226–37. 32238485 10.1542/neo.21-4-e226

[18] Yallapragada S, Savani RC, Munoz-Blanco S, Lagatta JM, Truog WE, Porta NFM, et al. Qualitative indications for tracheostomy and chronic mechanical ventilation in patients with severe bronchopulmonary dysplasia. J Perinatol. 2021;41:2651–7. 34349231 10.1038/s41372-021-01165-9 PMC8331995

[19] Ramanathan R, Biniwale M. Noninvasive ventilation. Crit Care Nurs Clin North Am. 2024;36:51–67. 38296376 10.1016/j.cnc.2023.11.001

[20] Nzegwu NI, Mack T, DellaVentura R, Dunphy L, Koval N, Levit O, et al. Systematic use of the RAM nasal cannula in the Yale-New Haven Children’s Hospital Neonatal Intensive Care Unit: a quality improvement project. J Matern Fetal Neonatal Med. 2015;28:718–21. 24874561 10.3109/14767058.2014.929659

[21] Biniwale M, Wertheimer F. Decrease in delivery room intubation rates after use of nasal intermittent positive pressure ventilation in the delivery room for resuscitation of very low birth weight infants. Resuscitation. 2017;116:33–38. 28476473 10.1016/j.resuscitation.2017.05.004

[22] Matlock DN, Ratcliffe SJ, Courtney SE, Kirpalani H, Firestone K, Stein H, et al. The Diaphragmatic Initiated Ventilatory Assist (DIVA) trial: study protocol for a randomized controlled trial comparing rates of extubation failure in extremely premature infants undergoing extubation to non-invasive neurally adjusted ventilatory assist versus non-synchronized nasal intermittent positive pressure ventilation. Trials. 2024;25:201. 38509583 10.1186/s13063-024-08038-4 PMC10953115

[23] Gerdes JS, Sivieri EM, Abbasi S. Factors influencing delivered mean airway pressure during nasal CPAP with the RAM cannula. Pediatr Pulmonol. 2016;51:60–9. 25851534 10.1002/ppul.23197

[24] Owen LS, Morley CJ, Dawson JA, Davis PG. Effects of non-synchronised nasal intermittent positive pressure ventilation on spontaneous breathing in preterm infants. Arch Dis Child Fetal Neonatal Ed. 2011;96:F422–428. 21335623 10.1136/adc.2010.205195

[25] Matlock DN, Bai S, Weisner MD, Comtois N, Beck J, Sinderby C, et al. Tidal volume transmission during non-synchronized nasal intermittent positive pressure ventilation via RAM((R)) cannula. J Perinatol. 2019;39:723–9. 30755718 10.1038/s41372-019-0333-x

[26] Lynch AL, Matlock DN, Akmyradov C, Weisner MD, Beck J, Sinderby C, et al. Tidal volume delivery during nasal intermittent positive pressure ventilation: infant cannula vs. nasal continuous positive airway pressure prongs. J Perinatol. 2024;44:244–9. 38129599 10.1038/s41372-023-01846-7

[27] Kamerkar A, Hotz J, Morzov R, Newth CJL, Ross PA, Khemani RG. Comparison of effort of breathing for infants on nasal modes of respiratory support. J Pediatr. 2017;185:26–32 e23. 28366356 10.1016/j.jpeds.2017.02.060 PMC5529226

[28] Estay AS, Mariani GL, Alvarez CA, Milet B, Agost D, Avila CP, et al. Randomized controlled trial of nonsynchronized nasal intermittent positive pressure ventilation versus nasal CPAP after extubation of VLBW infants. Neonatology. 2020;117:193–9. 32388511 10.1159/000506164

