# Supplementary material for: Nasal intermittent positive pressure ventilation in neonates with grade 3 bronchopulmonary dysplasia
Source: J Perinatol. 2025 Nov 17;46(2):193–9. doi: 10.1038/s41372-025-02472-1 (PMC12909103; doi:10.1038/s41372-025-02472-1)
Supplement: Supplementary file 1 — Center for Lung Development Clinical Practice Guidelines [file 41372_2025_2472_MOESM1_ESM.pdf]

Center for Lung Development Clinical Practice Guidelines

Updated 1/24/2023

Non-invasive ventilation

1. 36-42 weeks PMA

   Disclaimer: There are no evidence-based recommendations for non-synchronized NIPPV in sBPD patients. The following recommendations are based on local experience.

   a. Extubate to non-synchronized NIPPV
      i. PIP: start 30cm H_2O, max 40cm H_2O
      ii. PEEP: start 2-3cm H_2O over previous intubated PEEP, max 12
      iii. Rate: 40-50
      iv. iTime: 0.5-0.6
2. >42 weeks PMA
   a. Extubate to flow-triggered NIPPV
      i. PIP: start 30-35cm H_2O, max 45cm H_2O
      ii. PEEP: start 2-3cm H_2O over previous intubated PEEP, max 14
      iii. PS: 50% ΔP
      iv. Rate: 30-40
      v. iTime: 0.6-1
3. Alternative strategies
   a. Consider NIV-NAVA for improved synchronization
   b. Consider increasing PEEP above recommended limits if needed for airway disease

Adjusting NIPPV in patients with severe BPD

1. Weaning criteria (same as on SIMV)
   a. HCO_3 ≤32 (or PCO_2 ≤60 if unable to measure HCO_3)
   b. FiO_2 ≤50%
   c. Pro-growth state
      i. Meeting growth goals
      ii. Tolerating adequate nutrition and protein
      iii. Minimal environmental stress
      iv. Meeting goal weight gain
2. Ensure RAM cannula size is adjusted as patient grows
3. Wean PIP ~3cm H_2O each week, as tolerated
4. Wean PIP first and then Rate and lastly PEEP

Transition to CPAP

1. Ready for CPAP when stable for 3-4 days on PIP <10 over PEEP
2. Wean rate by 10 no faster than every 6 hours
3. Wean from rate 10 to CPAP at previous PEEP

Criteria for Re-intubation (extubation failure)

1. Early criteria (1st 72 hours)
    a. Saturation persistently <92% (on up to 100% FiO_2)
    b. Uncompensated respiratory acidosis
    c. Significant respiratory distress and/or stridor
2. Late criteria (>72 hours)
    a. FiO_2 >70% to maintain saturation >92%
    b. Rising serum HCO_3 levels
    c. Growth failure (persistent Z-score decline after optimization of calories and protein: length decline >1 and/or weight decline >0.5)
3. Very late criteria (>8 weeks)
    a. Failure to meet weaning criteria consistently for at least 8 weeks.
    b. Growth failure as defined above
